# The Surplus Effect in Adaptive Behaviour in Down Syndrome: What Can Promote It?

**DOI:** 10.3390/brainsci11091188

**Published:** 2021-09-10

**Authors:** Anastasia Dressler, Valetina Perelli, Margherita Bozza, Stefania Bargagna, Franz Benninger, Anna Kosheleva, Eva Schernhammer

**Affiliations:** 1 Division of Neonatology, Paediatric Intensive Care and Neuropaediatrics, Department of Paediatrics and Adolescent Medicine, Medical University of Vienna, 1090 Vienna, Austria; 2 IRCCS, Stella Maris Foundation, Scientific Institute for Child and Adolescence Neurology and Psychiatry, University of Pisa, 56126 Pisa, Italy; v.perelli@fsm.unipi.it (V.P.); drssamargheritabozza@gmail.com (M.B.); s.bargagna@fsm.unipi.it (S.B.); 3 Department of Child Psychiatry, Medical University of Vienna, 1090 Vienna, Austria; franz.benninger@meduniwien.ac.at; 4 Centre of Public Health, Department of Epidemiology, Medical University of Vienna, 1090 Vienna, Austria; akoshele@hsph.harvard.edu (A.K.); eva.schernhammer@meduniwien.ac.at (E.S.)

**Keywords:** Down syndrome, adaptive behaviour, surplus effect, Vineland Adaptive Behaviour Scales, inclusive schooling, early treatment programmes, parental education

## Abstract

Background: In Down syndrome (DS), adaptive behaviour often shows a “surplus effect” (i.e., higher adaptive abilities than expected from cognitive skills). As inclusive schooling has become mandatory in Italy, we studied the impact of school inclusion on the surplus effect of adaptive behaviour in adult DS, considering potential confounding factors such as parental education. Methods: All consecutive DS individuals from three different sites were queried prospectively regarding type of schooling (inclusive and non-inclusive). Demographic data were documented; cognitive abilities and adaptive behaviour were assessed (Coloured Progressive Matrices and Vineland Adaptive Behaviour Scales). The aim was to establish the presence of a surplus effect in adaptive behaviour, primarily in the overall level and secondarily in the main domains and subdomains. A multivariable-adjusted logistic regression model was used for the association of schooling, and parental education. Results: The majority (65%) showed a surplus effect in adaptive behaviour and had attended inclusive schools (85%). Higher adaptive skills as well as early and longer functional treatment programmes were more readily available for younger individuals. In the group of inclusive schooling, the surplus effect on overall adaptive behaviour was present in 70% as opposed to 38% in the group without inclusive schooling, significant when adjusted for gender and maternal education. This was also observed in socialisation, written, and community, and after adjustment in playing and leisure time. Conclusions: Adaptive behaviour showed a surplus effect in the majority of DS adults, even more so after inclusive schooling. Younger adults showed higher adaptive skills. Moreover, female gender and higher maternal educational level significantly enhanced this surplus effect.

## 1. Introduction

Adaptive behaviour is defined as “the effectiveness with which the individual copes with the natural and social demands of his environment” [1]. Such skills in daily functioning are essential for personal and social autonomy [2] and are particularly crucial for individuals with intellectual disabilities, (ID) when cognitive testing is difficult [3], allowing us to evaluate their mastery of the daily environment.

Vianello et al. [4] defined the “surplus effect” as performance above the average compared to the expected potential on the basis of mental age. However, daily life performance in ID is not only determined by intrinsic factors such as cognitive and linguistic abilities, as motivation and efficacy are also influenced by external factors. The presence of strangers, for example, and the dependence on a familiar adult person have been found to exert a negative influence [5], whereas educational programmes and inclusive schooling have been shown to foster the surplus effect [4,6,7,8].

For genetic syndromes such as DS, a large variability in adaptive behaviour exists [9], indicating that development is not only determined by genetics, but also by other factors (e.g., early, tailored rehabilitative programs, schooling, and occupational programmes [10]). Adaptive behaviour in individuals with Down syndrome (DS) typically shows a specific phenotypic profile with points of strength in self-care, daily living skills, and socialisation, and a point of weakness in reception [11]: adaptive skills are generally higher than cognitive and language abilities, and they continued to improve with age [12,13], even past the time when cognitive abilities have usually reached a plateau [14].

The few previous studies that examined the effect of early treatment programmes in childhood [15] on DS outcomes showed no early change in adaptive skills in childhood, and slowly increasing adaptive skills until middle adulthood [14]. In September 1978 (Legge no. 517/1977), inclusive schooling started to become mandatory for all children in Italy, obliging teachers to develop specific and individual treatment and educational programmes for each child with ID. Furthermore, the presence of a support teacher with the child in class also became mandatory.

In the current analysis, we sought to study the impact of inclusive schooling, parental educational levels, and early treatment programmes on adaptive functioning in DS adulthood. We hypothesised that school inclusion promotes the surplus effect of adaptive behaviour in DS adulthood. Secondarily, we hypothesised that age, early treatment programmes, and parental educational levels would additionally enhance the surplus effect.

## 2. Methods

### 2.1. Study Population

Between 2002 and 2007, we enrolled all consecutive individuals with clinical DS from an on-going prospective study on premorbid signs of Alzheimer’s disease in DS at the IRCCS Stella Maris Institute, Division of Child Neurology and Psychiatry, University of Pisa Italy (financed by the Italian Health Ministry RF 05/00) with three different sites included (Pisa and Livorno, Pistoia, Bologna) [14].

Inclusion criteria were living in the family as well as written informed consent, while exclusion criteria consisted of the presence of dementia, uncorrected metabolic disorders and uncontrolled seizures. All individuals were interviewed along with their main caregiver, and a complete medical history, a detailed medical examination including a neurological exam and a semi-structured psychiatric interview were performed. The following demographic variables were collected: gender, type of schooling (inclusive, specialised, none), years of schooling (for each type), age at start of schooling (for each type), parental and maternal years of education, age at onset of speech therapy, type, and duration of functional treatment (speech therapy, psychomotor therapy, and pedagogic therapy) and current day time activity outside home in days per week.

### 2.2. Outcome Assessment

Adaptive functioning was assessed with the Italian version of the Vineland Adaptive Behaviour Scales (VABS) as a structured interview with the caregivers [16]. The VABS total score for the Adaptive Behaviour Composite was calculated. We used age-equivalent scores for comparisons with mental age. For the calculation of areas of strength and areas of weakness, VABS raw scores were converted into three levels of functioning (above average, average, below average) according to the average of the measured cognitive level in this ID group, as previously published [14] and shown in Table 1. This is recommended by the authors [16] for the comparison of different levels of adaptive functioning, independent from cognitive performance, to illustrate points of strength, which are defined as the surplus effect. Therefore, individuals showing an overall level above the average for their cognitive level were defined as showing a surplus effect (yes) while those with an overall level on average or below their cognitive level were characterised as not showing a surplus effect (no). Cognitive abilities were assessed using the Raven’s Coloured Progressive Matrices (CPM), and the severity of intellectual disability (ID) was classified using the criteria of the “International Classification of Diseases [17].

### 2.3. Statistical Analyses

Data were compared between DS individuals with inclusive schooling during their childhood versus DS individuals without inclusive schooling. Descriptive parameters analysed were age (in years), gender, mental age (in years), individual schooling (in years), paternal and maternal educational levels (in years) and age at start, and duration of early functional treatments (in years). The primary endpoint was the presence of a surplus effect (performing above the average as expected for mental age) of the level of adaptive behaviour composite (overall level of adaptive behaviour). Secondary endpoints were the presence of a surplus effect in the main domains and subdomain levels of adaptive behaviour. For descriptive statistics, frequencies, mean or median, standard deviations, and minimum and maximum values were reported. For comparisons between groups, Pearson’s chi-square and Fisher’s exact test were applied. We used multivariable logistic regression models to adjust for age, gender, paternal and maternal educational level to examine associations between schooling type (inclusive yes versus no as the reference group) and surplus effect (binary; yes versus no).

All data were analysed using the IBM Statistical Package for Social Science (SPSS Statistics Version 23). Logistic regression was performed using SAS 9.4 (SAS Institute Inc., Cary, NC, USA). The significance level was set at *p* ≤ 0.05.

Ethic committee approval was obtained from the national research ethic committee of the Stella Maris Institute Pisa for the study (RF 05/00).

## 3. Results

### 3.1. Participants’ Characteristics

Fifty-four DS individuals were included (22 females) with a mean age of 28.6 ± 8.8 (min. 19 to max. 52.3 years). Thirty-five individuals were under 30 years old, while the remaining 19 participants were all above 30 years of age. All individuals showed the clinical phenotype of DS; in 51 individuals, clinical diagnosis was confirmed by cytogenetic analysis where 43 individuals (79.6%) showed complete Trisomy 21, and eight individuals (14.8%) showed mosaic DS. Cytogenetic analysis could not be performed on the three remaining individuals (5.6%) because of their refusal of blood withdrawal.

**Figure 1:**
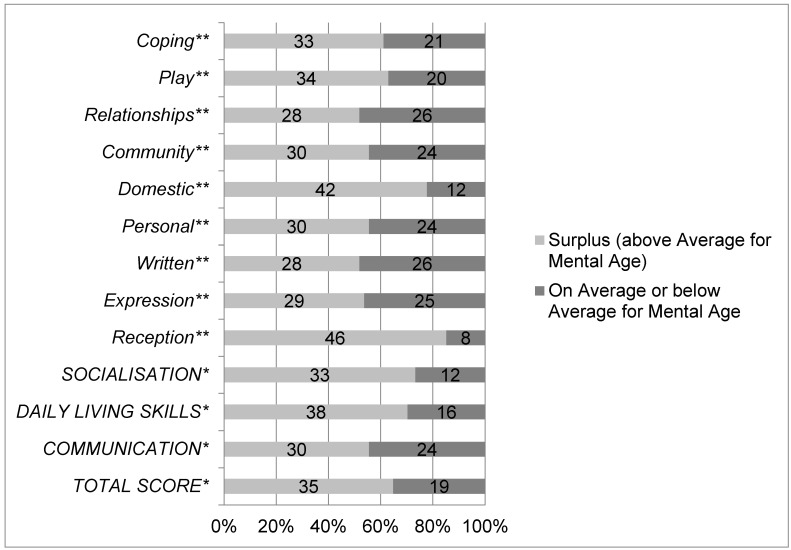
Proportion of individuals with DS (*n* = 52) who do (light grey) or do not (dark grey) exhibit a surplus effect on main domains * (communication, daily living skills, socialisation) and subdomains ** of adaptive behaviour (reception, expression, written, personal, domestic, community, relationships, play, and coping) and the adaptive behaviour composite * (total score). * Main domains and total score are written in capital letters. ** Subdomains are written in italic letters.

The majority of individuals showed a surplus effect regarding their overall level of adaptive behaviour (35/54, 65%) as well as in all the main domains and subdomains (Figure 1). The main domain of socialisation showed the highest mean equivalent age (Table 1). Within the subdomains, the highest mean equivalent ages were seen in domestic skills and coping strategies (mean 9.9 ± 3.3, min. 3.3–max. 16.9; mean 8.3 ± 3.6, min. 3.0–max. 15.3, respectively). Cognitive testing by CPM (in one individual with Leiter Scales) could be performed in 48 individuals. The remaining six participants were not testable by CPM or Leiter Scales and were classified as profound ID. By virtue of the small numbers in the profound and severe ID groups, these two groups were put together. Individuals were hence divided into three groups of ID (mild, moderate, and severe ID). The mean intelligence quotient (IQ) was 51.3 ± 12.2 (min. 30.0, max. 80.0); mean equivalent mental age at the 50th percentile was 5.2 ± 1.8 (min. 3.0; max 10.0 years). Seventeen individuals belonged to the mild ID-group (32%), 19 (35%) to the moderate ID-group, and 18 (33%) to the severe ID-group.

In total, 46/54 (85%) participants attended inclusive schools: 42/54 (77%) with immediate inclusion at school start, and 4/54 (7%) children during the last two years of elementary school. The remaining 8/54 (15%) did not attend inclusive schools: five (9%) never attended any school or treatment program, and three (6%) attended special schools.

Table 2 shows the relevant baseline characteristics split for groups with and without inclusive schooling. Statistically significant differences were observed for age, mental age, total school years, years of maternal and paternal schooling, and the duration of speech and of psychomotor therapy. With respect to these factors, participants with inclusive schooling were significantly younger, showing a substantially higher mental age and a remarkably higher number of attended school years for themselves as well as for both parents, and a significantly higher number of years of speech and psychomotor therapy (Table 2).

Table 3 shows the primary and secondary endpoints comparing participants with inclusive schooling with participants without inclusive schooling.

### 3.2. Primary Endpoint

For the overall level of adaptive behaviour, we observed a higher percentage of participants with a surplus in individuals with inclusive schooling as opposed to those without inclusive schooling (69.5% vs. 37.5%; *p* = 0.090), probably not significant due to the small sample size (Table 3).

The percentage of the surplus effect was higher in females than in males (73% versus 59%; *p* = 0.391, odds ratio 0.548 CI 0.17–1.8), however, not significantly. When looking at participants without inclusive schooling (five females and three males), three females showed a surplus effect in adaptive behaviour, while no male participant displayed this characteristic (*p* = 0.196). However, the significance can be interpreted ambiguously due to the small sample size. When comparing males with inclusive schooling to males without inclusive schooling, 65% of males with inclusive schooling showed a surplus effect while none of the males without inclusive schooling adhered to the pattern (*p* = 0.058).

Therefore, we adjusted the multivariate logistic regression model for (1) gender, (2) gender and paternal education (data not shown), and (3) gender and maternal education (Table 3).

The surplus effect in the DS individuals with inclusive schooling reached statistical significance adjusted for gender (*p* = 0.03) and adjusted for gender and for maternal educational level (*p* = 0.039), and showed a trend adjusted for gender and for paternal education (*p* = 0.085).

### 3.3. Secondary Endpoints

Secondary endpoints were the proportions of a surplus effect in the main domains and subdomains of adaptive behaviour in DS individuals with and without inclusive schooling. Individuals with inclusive schooling showed higher proportions of a surplus effect in all main domains and subdomains except in domestic skills. Significantly higher percentages of the surplus effect were observed for individuals with inclusive schooling in the main domain socialisation (*p* = 0.045), and in the subdomains written (*p* = 0.022) and community (*p* = 0.016).

After adjustment (Table 3) in the multivariate logistic regression model, the surplus effect in the domain socialisation showed a trend pertaining to gender (*p* = 0.077), and was significant in the subdomain community adjusted for gender (*p* = 0.023), for gender and paternal educational level (*p* = 0.039), and for gender and maternal educational level (*p* = 0.025). In the subdomain written, no significant differences were observed in the multivariate regression model. However, after adjustment, a higher percentage of surplus effect was observed in the subdomain play when adjusted for gender (*p* = 0.036) as well as for gender and maternal education (*p* = 0.051).

## 4. Discussion

In our study, we investigated the influence of inclusive schooling in childhood on the surplus effect of adaptive behaviour in DS adults as well as factors promoting this surplus effect. Adaptive behaviour in DS has been described as being higher than could be expected from cognitive abilities [6,18,19]. This phenomenon, first defined by Vianello and co-workers [6,18] as the surplus effect, does not only indicate a syndrome-specific phenotype, but also reveals how appropriate educational interventions and therapy programmes facilitate above average individual performance [4].

In the past, we found that DS-individuals with mild to moderate levels of ID performed significantly more often above the average than individuals with severe ID [14]. However, age equivalents of adaptive behaviour were higher than mental age in all age-groups. To study adaptive behaviour without the effect of cognition, the comparison of adaptive levels above the average (surplus effect) were referenced within their own ID-group.

The majority of DS individuals attended inclusive schools and showed a surplus effect in adaptive behaviour on the overall adaptive behaviour level as well as on all the main domains and subdomains. DS adults with inclusive schooling were significantly younger, showed a higher mental age, had attended school for a longer period of time, and had attended a higher number of years in early treatment programmes than DS adults having attended no inclusive schooling (i.e., special schooling) or even no schooling at all. Moreover, parental educational levels were significantly higher in individuals with inclusive schooling. Furthermore, female gender and parental, especially maternal educational levels significantly enhanced the surplus effect.

Limitations of our study are that adaptive behaviour data are cross-sectional without individual trajectories, and that, because of the mandatory school inclusion in Italy for every child since 1977, the group of individuals with inclusive schooling was higher than the group without inclusive schooling. Strengths are the high number of included DS adults and the comparison between inclusive and non-inclusive schooling.

We confirmed the surplus effect in all adaptive domains in our DS-cohort [11,14,18,19,20,21], but to our knowledge, this is the first study to describe that this surplus effect was higher in individuals with inclusive schooling, further enhanced by female gender and maternal educational level. This result was observed in the overall adaptive level as well as in the subdomains of community and play. The surplus effect on the overall adaptive behaviour level was present in 70% of the individuals with inclusive schooling vs. 38% without inclusive schooling, significant after adjustment for gender as well as for gender and maternal educational level. In the main domain of socialisation, the subdomains written and community, the surplus effect was significantly more frequent in individuals with inclusive schooling (68% vs. 25%; 59% vs. 13%; 63% vs. 13%; respectively). After adjustment for gender, for gender and paternal educational level as well as for gender and maternal educational level, these effects were still observed for the subdomain community as well as for the subdomain playing and leisure time.

In contrast to other European countries, Italy promoted the attendance of DS-individuals in mainstream schools with legally obligated support teachers since 1977, and the abolishment of special schools. Bertoli and co-workers [22] showed that reading skills in DS-individuals from 4–40 years of age starting school after 1971 were higher, when compared to DS-individuals entering school before 1971. In our cohort, we also confirmed this generational effect due to school inclusion, increases in parental educational levels and early treatment programmes. The literature has shown that school inclusion has benefitted individuals with ID with or without DS [23,24,25]. Buckley and co-workers observed higher scores on speech, language abilities, and academic activities in the 1999 group compared to the 1987 group, explained by inclusion in regular educational settings [23,26,27,28]. This was also observed in an Australian group with DS-individuals born between 1971 and 1978 compared to historical data from the UK and the U.S. [29]. In the Netherlands, a shift toward school inclusion took place later in the 1990s, showing improved academic outcomes despite lower cognitive skills due to regular educational settings [30]. Self-help was acquired until young adulthood, whereas computer skills showed generational differences.

The high percentage of the surplus effect in all our DS-individuals indicates that this is syndrome-specific (i.e., a DS-phenotype) [4]. However, the presence of the surplus effect associated with inclusive schooling, younger age, an earlier start, and a higher duration of early treatment programmes confirms that the situation in Italy after 1977 has changed adaptive skills for the better [4,6,7,8,10].

To our knowledge, this is the first study to consider the effect of parental education on the surplus effect in DS, showing that parental, especially maternal, schooling further enhances a child‘s adaptive potential, which is also true for children without ID [31,32,33] and may outweigh the syndrome-specific surplus effect in DS.

Moreover, female gender as an intrinsic enhancer of adaptive behaviour has been described in the literature [34], but neither in DS [14] nor in Italian neuro-typical children [33]. However, female gender and parental educational levels have already been described as enhancers of academic performance [35,36] in neuro-typical development.

## 5. Conclusions

A surplus effect (i.e., a performance above the average on adaptive skills) has been shown in the majority of DS individuals, enhanced by inclusive schooling, female gender, and parental (especially maternal) educational levels. Higher adaptive skills and early and longer functional treatment programs were more readily available for younger individuals, indicating that obliging school inclusion and better early treatment programmes in Italy resulted in a striking generational difference of functional skills after 1977.

## Figures and Tables

**Table 1:** Mean equivalent ages of adaptive behaviour in DS individuals on main domains, total score and mean mental age (in years).

	Mean Age *	Standard Deviation	Minimum	Maximum
**Adaptive Skills**				
**Communication**	7.0	3.4	1.0	13.0
**Daily Living Skills**	7.3	2.6	2.3	13.0
**Socialisation**	**7.3**	**4.3**	**1.7**	**16.9**
**Total score**	7.1	3.2	1.7	13.9
**Mental age**	5.2	1.8	3.0	10.0

* Mean age in years. Bold numbers highlight the highest level of equivalent age in the domain socialisation.

**Table 2:** Patient characteristics in DS individuals who attended (light grey) or did not attend (white) inclusive schooling.

	Inclusive Schooling Yes (*n* = 46)	Inclusive Schooling No (*n* = 8)	Sig.
Age, in years *	26.2 ± 6.3	43 ± 7.3	**<0.001**
Female **	17 (37%)	5 (63%)	0.248
Mental age, in years *	5.5 ± 1.8	3.6 ± 0.8	**0.020**
Total school years *, ***	11.4 ± 2.3	1.5 ± 3	**<0.001**
Years of inclusive school *	10.3 ± 3.4	0 ± 0.0	**<0.001**
Mother’s years of schooling *	8.8 ± 4.7	2.6 ± 3.8	**0.001**
Father’s years of schooling *	8.8 ± 4.6	2.9 ± 2.5	**0.001**
Duration of speech therapy *	5.4 ± 4.2	0.3 ± 0.7	**0.001**
Duration of psychomotor therapy *	3.8 ± 4.2	0.5 ± 1.4	**0.034**
Duration of pedagogic therapy *	2.4 ± 4.4	0.6 ± 1.8	0.262
Current day time activity outside of home, days/week *	4.4 ± 1.5	4.4 ± 1.8	0.919
Living in a city > 100.000 dwellers **	19 (41%)	2 (25%)	0.383
Living in a city < 100.000 dwellers **	27 (59%)	6 (75%)	0.383

* mean, SD. ** *n*, %; For frequencies with Chi-square and Fisher’s exact test were used. *** Including, inclusive, and “special” school years. Bold numbers highlight significance level *p* < 0.001.

**Table 3:** Proportion of DS individuals with a surplus effect * in adaptive behaviour divided by inclusive and non-inclusive schooling (adjusted for gender and maternal education by regression).

	Number of Individuals with SURPLUS *, *n* (%)	Odds Ratio	95% CI	*p*-Value	Odds Ratio MODEL 1 (gender)	95% CI	*p*-Value	Odds Ratio MODEL 3 (Gender & Maternal Education)	95% CI	*p*-Value
**Adaptive Behaviour**	Inclusive schooling Yes (*n* = 46)/ No (*n* = 8)									
**Total Score**	32 (70%)/3 (28%)	3.8	0.8–18	**0.090**	16.4	1.3–206	**0.030**	**14.9**	1.1–194	**0.039**
**Main Domains**										
Communication	27 (59%)/3 (38%)	2.4	0.5–11	0.443	4.1	0.5–35.5	0.203	3.6	0.4–32.9	0.256
Daily Living Skills	33 (72%)/5 (63%)	1.5	0.3–7	0.682	3.9	0.4–40.8	0.255	3.9	0.4–41.9	0.682
Socialisation	31 (68%)/2 (25%)	**6.2**	1.1–34	**0.045**	8.9	0.8–100	**0.077**	7.7	0.6–91.6	0.106
**Subdomains**										
Reception	40 (87%)/6 (75%)	2.2	0.3–13	0.588	0.9	0.1–12.2	0.908	0.8	0.06–11.6	0.873
Expression	26 (57%)/3 (38%)	2.2	0.9–10	0.449	1.7	0.2–13.9	0.629	1.1	0.1–11.2	0.449
Written	27 (59%)/1 (13%)	**9.9**	1.1–87	**0.022**	9.1	0.6–129	0.102	7.4	0.5–114	0.150
Personal Skills	26 (57%)/4 (50%)	1.3	0.3–6	1.000	1.8	0.2–14.5	0.596	1.6	0.2–13.6	0.656
Domestic Skills	35 (76%)/7 (88%)	0.5	0.5–4	0.667	0.5	0.03–7.4	0.583	0.4	0.02–6.6	0.520
Community	29 (63%)/1 (13%)	**11.9**	1.3–105	**0.016**	**28.4**	1.6–506	**0.023**	**27.7**	1.5–500	**0.016**
Interpersonal Relations	26 (57%)/4 (50%)	3.9	0.7–21	0.135	3.3	0.3–30.8	0.328	2.7	0.3–27.5	0.401
Play	31 (67%)/3 (38%)	3.4	0.7–16	0.130	**14.4**	1.2–172	**0.036**	**12.9**	1.0–169	**0.050**
Coping skills	29 (63%)/4 (50%)	1.7	0.4–7	0.697	1.3	0.2–10.4	0.823	0.9	0.1–8.4	0.943

* Surplus has been defined as the effect when an individual performs above the average as compared to the expected potential on the basis of mental age (Vianello et al., 2006). Proportions were calculated by Fisher’s exact test. Bold numbers highlight significant odds ratios and significance level *p* < 0.050. Light grey indicate percentages of inclusive schooling. Description: The primary endpoint was adjusted in a multivariate logistic regression model for (1) gender (model 1), (2) gender and paternal education (model 2) (data not shown), and (3) gender and maternal education (model 3). Adjusted for gender, DS individuals with inclusive schooling showed 16.4 times higher odds for a surplus effect on the total score of adaptive behaviour (primary endpoint); adjusted for gender and maternal education DS individuals with inclusive schooling showed 14.9 higher odds. Adjusted for gender, DS individuals with inclusive schooling showed 28.4 times higher odds for a surplus effect in the subdomain community and 14.4 times higher odds in the subdomain play. This was also observed when adjusted for gender and paternal education in the subdomain community (OR 22.3) and when adjusted for gender and maternal education (Table 3) in the subdomains community and play (Table 3).

## Data Availability

The data presented in this study are available on request from the corresponding author. The data are not publicly available due to privacy and ethical reasons.
